# Author Correction: Cretaceous bird from Brazil informs the evolution of the avian skull and brain

**DOI:** 10.1038/s41586-026-10960-3

**Published:** 2026-08-13

**Authors:** Luis M. Chiappe, Guillermo Navalón, Agustín G. Martinelli, Ismar de Souza Carvalho, Rodrigo Miloni Santucci, Yun-Hsin Wu, Daniel J. Field

**Affiliations:** 1 Dinosaur Institute, Natural History Museum of Los Angeles County, Los Angeles, CA USA; 2 Department of Earth Sciences, University of Cambridge, Cambridge, UK; 3 Department of Life Sciences, Global Change Ecology and Evolution Research Group (GloCEE), Universidad de Alcalá, Madrid, Spain; 4 Sección Paleontología de Vertebrados, CONICET—Museo Argentino de Ciencias Naturales Bernardino Rivadavia, Buenos Aires, Argentina; 5 Instituto de Geociências, Universidade Federal do Rio de Janeiro, Rio de Janeiro, Brazil; 6 Centro de Geociências, Coimbra University, Coimbra, Portugal; 7 Faculdade UnB Planaltina, Universidade de Brasília, Brasília, Brazil; 8 Museum of Zoology, University of Cambridge, Cambridge, UK

**Keywords:** Taxonomy, Herpetology

Correction to: *Nature* https://doi.org/10.1038/s41586-024-08114-4 Published online 30 October 2024

In the originally published version of our article, the endocranial landmark configuration from the enantiornithine bird *Navaornis hestiae* included nine misplaced patch semilandmarks resulting from a sharp corner in the three-dimensional model, which inadvertently shifted those semilandmarks to the next solid surface along the landmark’s normal trajectory. Here, we amend this problem by repositioning these misplaced coordinates using the software Stratovan Checkpoint (the state-of-the-art licensed and maintained version of the software used in our original manuscript: IDAV Landmark Editor) and upload the corrected dataset to the link provided in the Data availability section in the original article. We produced an updated principal components plot summarizing patterns of endocranial shape with the corrected dataset (Fig. 1). Patterns are unchanged regarding the ‘intermediate’ morphology of *N. hestiae* along PC1, the main axis of endocranial shape variation. However, with the corrected dataset, *N. hestiae* falls within the crown bird dispersion along PC2, whereas in the initially published article, *N. hestiae* fell just outside crown bird variation along the same axis. The amount of shape variation explained by each principal component in the corrected dataset varies slightly from the values in the original article; however, our interpretations of avian endocranial evolution are unaltered by this relatively minor change. To further reassess our initial interpretations, we calculated total shape distances (Procrustes distances) from *Navaornis hestiae* to all the taxa included in our sample (Fig. 2). The six closest species in endocranial shape include the stem bird *Archaeopteryx*, the unnamed troodontid IGM 100/1126, and crown birds such as *Ptilinopus* (Columbiformes), *Cariama* (Cariamiformes), *Crypturellus* (Palaeognathae, Tinamiformes) and *Caloenas* (Columbiformes). This assortment of taxa highlights the combination of plesiomorphic and crown bird-like traits in the endocranial morphology of *N. hestiae*, as originally interpreted. We thank Jesús Marugán-Lobón and Jingmai O’Connor, whose queries helped us identify issues with the original dataset. We also thank Patrick O’Connor and three anonymous reviewers who helped us improve the text of this correction notice.

**Fig. 1:**
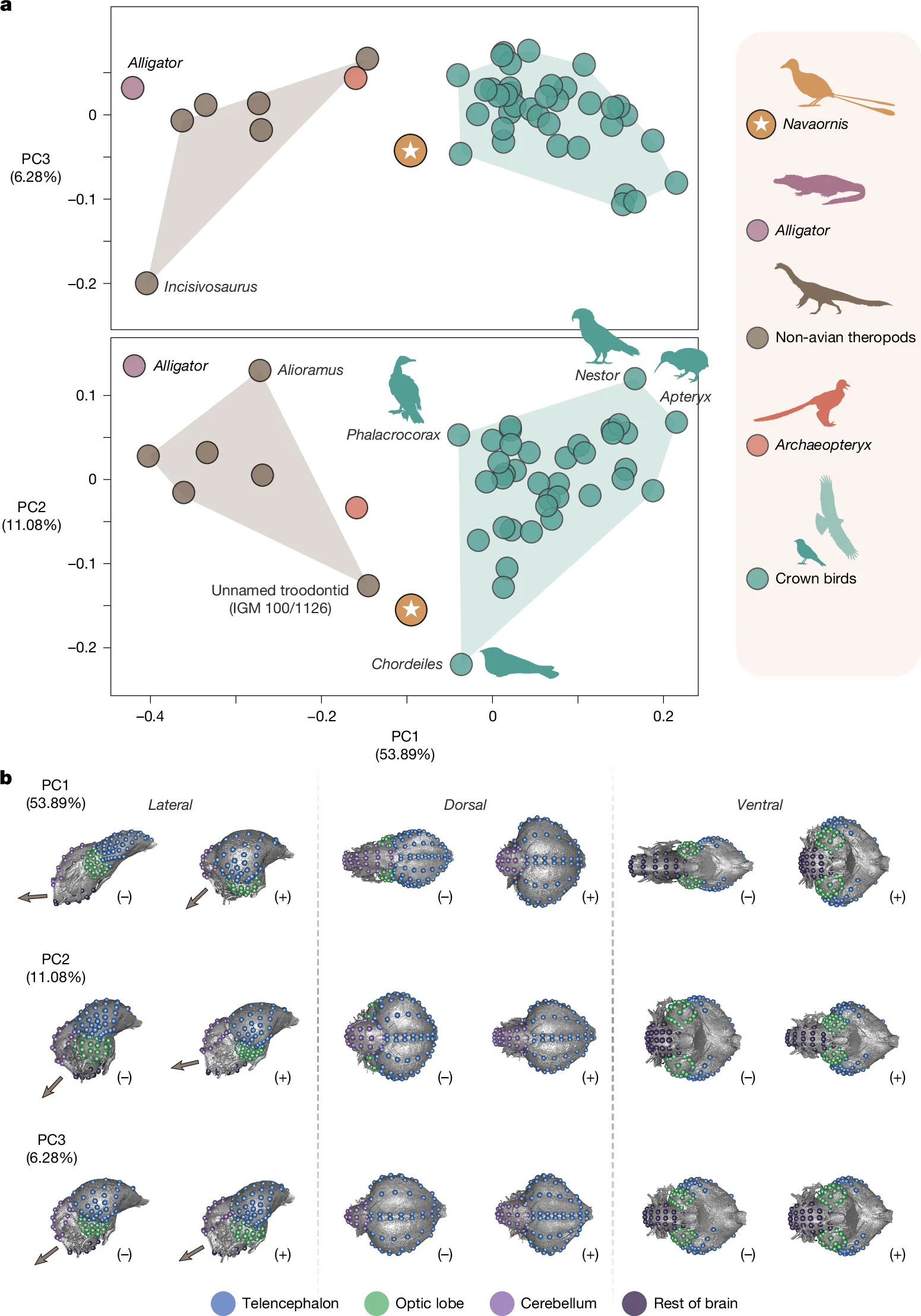
*Navaornis* and endocranial evolution in birds and non-avian dinosaurs. (**a**) Three-dimensional principal component (PC) morphospace (PC1 versus PC2, and PC1 versus PC3) of cranial endocasts from crown birds, *Archaeopteryx*, *Navaornis* and relevant non-avian taxa. Principal component plots are scaled to the same ratio, so distances are the same on the *x* axis and *y* axis. Along PC1, *Navaornis* falls in an intermediate position between stemward taxa such as *Archaeopteryx* and crown birds, whereas it falls within the range of crown bird variation along PC2 and PC3. (**b**) Shape changes associated with scores representing the 5th and 95th quantiles from each of the three main PCs of endocranial shape. Plus (+) and minus (−) symbols indicate whether shape warps are associated with positive or negative scores along each axis. Landmarks are colour-coded for the main brain regions. Brown arrows in **b** depict the foramen magnum orientation. Percentages in parentheses in **a** and **b** indicate the proportion of total shape variance explained by each of the principal components. This figure represents a correction of the PCA plots in Fig. 4b and Extended Data Fig. 10d from the original article.

**Fig. 2:**
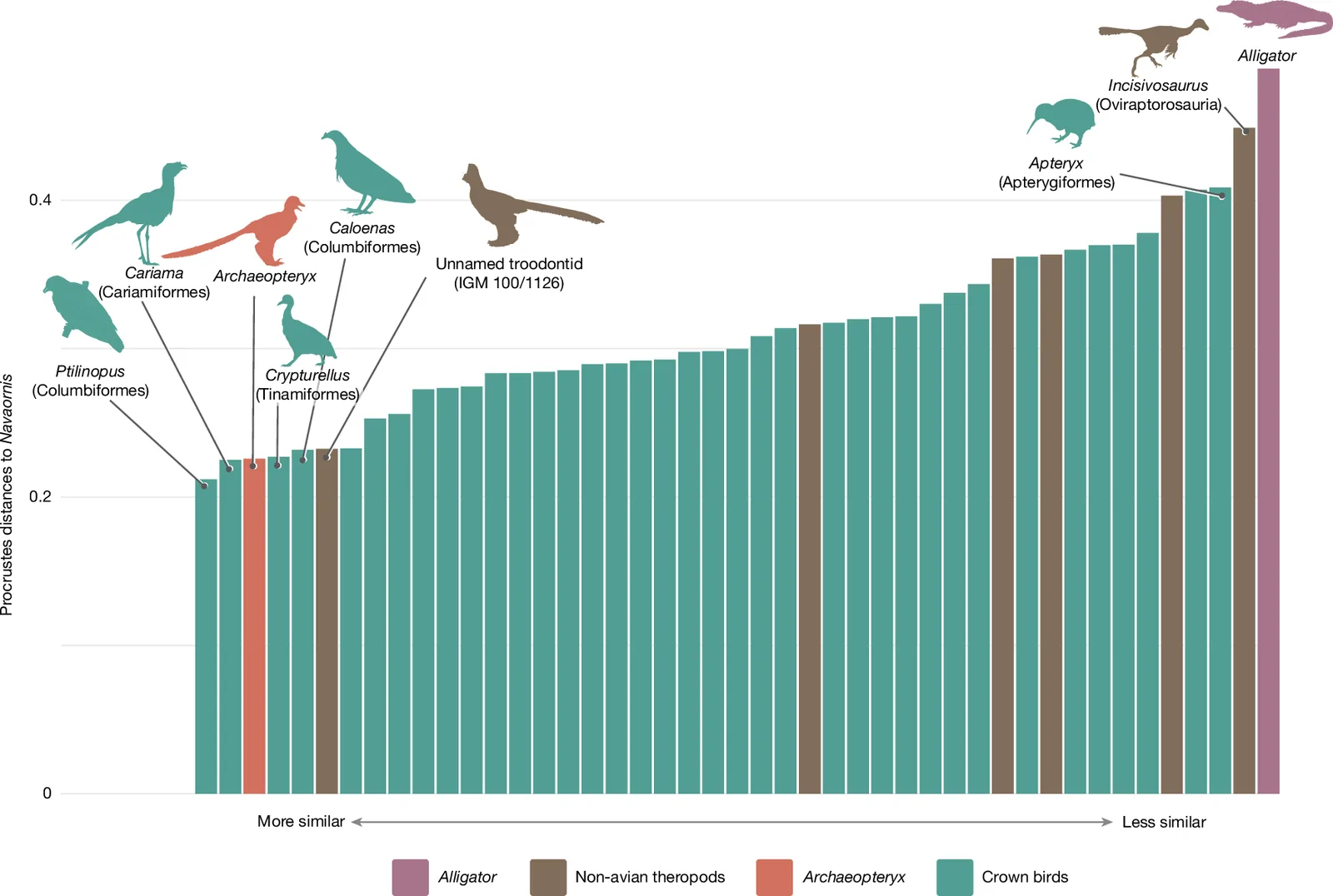
Endocranial shape similarity between individual crown and stem bird taxa and *Navaornis hestiae.* Shape differences are quantified as Procrustes distances between landmark configurations after Generalized Procrustes Superimposition (Procrustes residuals). Species values in the histogram plot are ordered from most similar to least similar. The individual taxa closest and furthest to *Navaornis* in endocranial shape are labelled. Note that among the shortest Procrustes distances relative to *Navaornis* are a handful of stem bird taxa as well as several crown birds, underlining the intermediate geometry of the *Navaornis* endocast between that of *Archaeopteryx* and the avian crown group. The assortment of taxa closest to *Navaornis* do not necessarily resemble *Navaornis* in the same aspects of their endocranial geometry.

