# Supplementary material for: Cretaceous bird from Brazil informs the evolution of the avian skull and brain
Source: Nature. 2024 Oct 30;635(8038):376–81. doi: 10.1038/s41586-024-08114-4 (PMC11560842; doi:10.1038/s41586-024-08114-4)
Supplement: Supplementary file 1 — Reporting Summary [file 41586_2024_8114_MOESM1_ESM.pdf]

nature portfolio

Corresponding author(s): Guillermo Navalón, Luis M. Chiappe and Daniel J. Field

Last updated by author(s): 08/08/2024

Reporting Summary

Nature Portfolio wishes to improve the reproducibility of the work that we publish. This form provides structure for consistency and transparency in reporting. For further information on Nature Portfolio policies, see our Editorial Policies and the Editorial Policy Checklist.

Statistics

For all statistical analyses, confirm that the following items are present in the figure legend, table legend, main text, or Methods section.

n/a	Confirmed	
☐	☒	The exact sample size (*n*) for each experimental group/condition, given as a discrete number and unit of measurement
☒	☐	A statement on whether measurements were taken from distinct samples or whether the same sample was measured repeatedly
☒	☐	The statistical test(s) used AND whether they are one- or two-sided<br>*Only common tests should be described solely by name; describe more complex techniques in the Methods section.*
☐	☒	A description of all covariates tested
☐	☒	A description of any assumptions or corrections, such as tests of normality and adjustment for multiple comparisons
☒	☐	A full description of the statistical parameters including central tendency (e.g. means) or other basic estimates (e.g. regression coefficient) AND variation (e.g. standard deviation) or associated estimates of uncertainty (e.g. confidence intervals)
☒	☐	For null hypothesis testing, the test statistic (e.g. *F*, *t*, *r*) with confidence intervals, effect sizes, degrees of freedom and *P* value noted<br>*Give P values as exact values whenever suitable.*
☒	☐	For Bayesian analysis, information on the choice of priors and Markov chain Monte Carlo settings
☒	☐	For hierarchical and complex designs, identification of the appropriate level for tests and full reporting of outcomes
☒	☐	Estimates of effect sizes (e.g. Cohen's *d*, Pearson's *r*), indicating how they were calculated

Our web collection on statistics for biologists contains articles on many of the points above.

Software and code

Policy information about availability of computer code

Data collection	MPM-200-1 (holotype specimen for Navaornis hestiae gen. nov. sp. nov.) was scanned using a Bruker SkyScan 1173 CT scanner at the Instituto de Petróleo e dos Recursos Naturais (Laboratório de Sedimentologia e Petrologia) of the Pontifícia Universidade Católica do Rio Grande do Sul (PUCRS), Porto Alegre, Rio Grande do Sul, Brazil.
Data analysis	MPM-200-1 was scanned using a Bruker SkyScan 1173 CT scanner at the Instituto de Petróleo e dos Recursos Naturais (Laboratório de Sedimentologia e Petrologia) of the Pontifícia Universidade Católica do Rio Grande do Sul (PUCRS), Porto Alegre, Rio Grande do Sul, Brazil. Scanning parameters were as follows: 10.71 µm voxel size, 60 kV, 133 µA, exposure time 850 ms, averaging two frames, no 360° rotation, using an aluminium filter of 1.0 mm. Slices were reconstructed using the software NRecon v.1.7.4.6.<br><br>Volumes were digitally reconstructed and segmented using VGSTUDIOMAX 3.4.0 (VolumeGraphics).<br><br>Three-dimensional meshes of each recognisable segmented cranial element and endocranial surface were exported from VGSTUDIOMAX 3.4.0 to Blender 3.3.0, where they were rearticulated to reconstruct the skull and endocranial anatomy of Navaornis hestiae.<br><br>Owing to the remarkable lack of distortion of most cranial elements, only the left frontal bone required slight retrodeformation following established best practices ion Blender 3.3.0.<br><br>Landmark-based geometric morphometrics were used to quantitatively compare the exocranial and endocranial anatomy of Navaornis with crown birds and a selection of non-avian dinosaurs and Mesozoic birds. Exocranial landmarks were digitised in Avizo Lite 2019.3 (Thermo Fisher Scientific), and endocranial landmarks were digitised in Landmark Editor following previously described procedures. Thereafter, the landmark datasets were imported into the R statistical environment v4.1.2, where all downstream analyses were conducted (complete R code

in Zenodo following this link: https://doi.org/10.5281/zenodo.10696014).

Phylogenetic analyses were conducted to ascertain phylogenetic relationships of the new taxon. Heuristic parsimony analyses were applied to a previously published and expanded (adding Navaornis and Yuornis) dataset using TNT v.1.670 under equal and implied weights (K = 3, K = 9, K =12).

For manuscripts utilizing custom algorithms or software that are central to the research but not yet described in published literature, software must be made available to editors and reviewers. We strongly encourage code deposition in a community repository (e.g. GitHub). See the Nature Portfolio guidelines for submitting code & software for further information.

Data

Policy information about availability of data

All manuscripts must include a data availability statement. This statement should provide the following information, where applicable:

- Accession codes, unique identifiers, or web links for publicly available datasets
- A description of any restrictions on data availability
- For clinical datasets or third party data, please ensure that the statement adheres to our policy

Scan data and surface meshes of all preserved elements of Navaornis are housed on MorphoSource (morphosource.org; https://www.morphosource.org/projects/000608371/temporary_link/2vM9BSS4HaYcq1R2eMcxbTpc?locale=en). Phylogenetic matrices and morphometric landmark coordinates are provided at Zenodo (https://doi.org/10.5281/zenodo.10696014).

Research involving human participants, their data, or biological material

Policy information about studies with human participants or human data. See also policy information about sex, gender (identity/presentation), and sexual orientation and race, ethnicity and racism.

Reporting on sex and gender	Not Applicable
Reporting on race, ethnicity, or other socially relevant groupings	Not Applicable
Population characteristics	Not Applicable
Recruitment	Not Applicable
Ethics oversight	Not Applicable

Note that full information on the approval of the study protocol must also be provided in the manuscript.

Field-specific reporting

Please select the one below that is the best fit for your research. If you are not sure, read the appropriate sections before making your selection.

☐ Life sciences ☐ Behavioural & social sciences ☒ Ecological, evolutionary & environmental sciences

For a reference copy of the document with all sections, see nature.com/documents/nr-reporting-summary-flat.pdf

Ecological, evolutionary & environmental sciences study design

All studies must disclose on these points even when the disclosure is negative.

Study description

We report on a new fossil bird species from the Late Cretaceous of Brazil, whose complete skull is exceptionally well-preserved in three dimensions. This enabled the complete description of both the external (skull) and, remarkably, internal (brain) cranial morphology of this remarkable new fossil, yielding long-sought insight into how and when the unique modern bird brain evolved.

Phylogenetic analyses resolve this new species as a member of the Mesozoic clade Enantiornithes, which split from the lineage leading to modern birds more than 130 million years ago.

Landmark-based geometric morphometrics were used to quantitatively compare the exocranial and endocranial anatomy of Navaornis with crown birds and a selection of non-avian dinosaurs and Mesozoic birds.

Our analyses allowed us to show that the skull of the new species displays an overall geometry that closely resembles that of modern birds, underscoring an unprecedented degree of convergence between these two distant avian lineages whereby archaic features of the skull acted as building blocks to yield a skull of surprisingly modern shape.

Importantly, we present the first detailed endocranial description of a stem bird crownward of Archaeopteryx, clarifying the pattern and timing by which the distinctive neuroanatomy of living birds was assembled.

Research sample	MPM-200-1 (Museu de Paleontologia de Marília, Marília, São Paulo State, Brazil). A complete skull (Fig.1) articulated with the anterior-most cervical vertebrae, extracted from a block (MPM-200; Extended Data Figure 1) from the Sítio Paleontológico de Presidente bonebed. A cast of MPM-200 has been accessioned at the Dinosaur Institute, Natural History Museum of Los Angeles County.
Sampling strategy	NA
Data collection	MPM-200-1 was scanned using a Bruker SkyScan 1173 CT scanner at the Instituto de Petróleo e dos Recursos Naturais (Laboratório de Sedimentologia e Petrologia) of the Pontifícia Universidade Católica do Rio Grande do Sul (PUCRS), Porto Alegre, Rio Grande do Sul, Brazil.<br><br>Three-dimensional meshes of each recognisable segmented cranial element and endocranial surface were exported from VGSTUDIOMAX 3.4.0 to Blender 3.3.0, where they were rearticulated to reconstruct the skull and endocranial anatomy of Navaornis hestiae . Owing to the remarkable lack of distortion of most cranial elements, only the left frontal bone required slight retrodeformation following established best practices. This was accomplished using the 'Lattice' function in Blender, where mediolateral compression of this element was corrected to match the geometry of the dorsal rim of the complete and mostly undistorted left parietal bone. The same degree of retrodeformation was then applied to the endocast surface of the left frontal, that is, the left hemisphere of the telencephalon. The remaining surfaces composing the endocranium of Navaornis hestiae were: ventral (derived from the basicrania of both the holotype and referred specimen MPM-334-1), anterior (derived from the right and left laterosphenoids of the holotype), dorsal and lateral surfaces of the left optic lobes (derived from the parietal of the holotype), cerebellum (derived from the parietal of the holotype and basicranium of both the holotype and referred specimen MPM-334-1), and medulla (derived from both the holotype and referred specimen MPM-334-1). Left and right surfaces were mirrored from the best-preserved element/endocranial surface.<br><br>Landmark-based geometric morphometrics were used to quantitatively compare the exocranial and endocranial anatomy of Navaornis with a selection of pre-existing published data from crown birds and a selection of non-avian dinosaurs and Mesozoic birds.<br><br>Specifically, Generalised Procrustes Analysis were performed on both sets of landmark coordinates to separate shape data from size and other confounding factors, and the minimum bending energy criterion was used to slide curve (exocranium and endocranium) and patch (endocranium) semilandmarks following previously described procedures, using the function 'gpagen' in the R package geomorph v.4.0.5.<br><br>Principal Components Analyses were carried out on the exocranial and endocranial Procrustes coordinates to visualise shape variation using the function 'gm.prcomp' in geomorph.<br><br>To determine the extant species geometrically closest to Navaornis in exocranial shape, we determined the Procrustes distances between Navaornis and all extant taxa in our dataset (Extended Data Figure. 9) using Euclidean distances with the function 'dist' from the R package stats v.4.1.2.<br><br>Changes associated with major axes of exocranial and endocranial shape variation were illustrated as deformations warped from the three-dimensional surface of the exocranium and endocranium of the individual species closest to the mean shape in both samples. Specifically, this three-dimensional surface and the mean shape from the sample were projected onto the scores representing the 0.05 and 0.95 quantiles for each PC axis by means of thin-plate spline deformation using the function 'tps3d' from the package Morpho v.2.10 and 'shape.predictor' from geomorph. We also plotted the respective landmark configurations onto the deformed meshes using 'shape.predictor', and coloured these landmark constellations according to per-landmark-variances from each dataset using the 'hot.dots' function (freely available following this link: https://zenodo.org/record/3929193).
Timing and spatial scale	Not Applicable
Data exclusions	Not Applicable
Reproducibility	All the raw data and the methods are reported to ensure complete reproducibility.
Randomization	Not Applicable
Blinding	Not Applicable

Did the study involve field work? ☐ Yes ☒ No

Reporting for specific materials, systems and methods

We require information from authors about some types of materials, experimental systems and methods used in many studies. Here, indicate whether each material, system or method listed is relevant to your study. If you are not sure if a list item applies to your research, read the appropriate section before selecting a response.

Materials & experimental systems

n/a	Involved in the study
☒	☐ Antibodies
☒	☐ Eukaryotic cell lines
☐	☒ Palaeontology and archaeology
☒	☐ Animals and other organisms
☒	☐ Clinical data
☒	☐ Dual use research of concern
☒	☐ Plants

Methods

n/a	Involved in the study
☒	☐ ChIP-seq
☒	☐ Flow cytometry
☒	☐ MRI-based neuroimaging

Palaeontology and Archaeology

Specimen provenance

Referred specimens. MPM-334-1, an isolated basicranium from the Sítio Paleontológico de Presidente Prudente. MPM-200 includes a partially articulated postcranial skeleton, which is also referred to Navaornis hestiae and hypothesised to represent the same individual as MPM-200-1 (see Extended Data Figure 1).

Locality and Age. William's Quarry, Sítio Paleontológico, Presidente Prudente, São Paulo State, Brazil. The quarry is contained within the Adamantina Formation (Bauru Group, Bauru Basin); various lines of evidence suggest a late Santonian to early Campanian age (~85-75 million years ago) for this site.

Specimen deposition

MPM-200-1 (Museu de Paleontologia de Marília, Marília, São Paulo State, Brazil).

Dating methods

William's Quarry, Sítio Paleontológico, Presidente Prudente, São Paulo State, Brazil. The quarry is contained within the Adamantina Formation (Bauru Group, Bauru Basin); various lines of evidence14-17 suggest a late Santonian to early Campanian age (~85-75 million years ago) for this site.

☒ Tick this box to confirm that the raw and calibrated dates are available in the paper or in Supplementary Information.

Ethics oversight

No ethical approval or guidance from a specific institution was followed.

Note that full information on the approval of the study protocol must also be provided in the manuscript.

Plants

Seed stocks

Not Applicable

Novel plant genotypes

Not Applicable

Authentication

Not Applicable
